# Improved Circuits with Capacitive Feedback for Readout Resistive Sensor Arrays

**DOI:** 10.3390/s16020149

**Published:** 2016-01-25

**Authors:** Óscar Oballe-Peinado, Fernando Vidal-Verdú, José A. Sánchez-Durán, Julián Castellanos-Ramos, José A. Hidalgo-López

**Affiliations:** 1 Departamento de Electrónica, Universidad de Málaga, Andalucía Tech, Campus de Teatinos, Málaga 29071, Spain; oballe@uma.es (O.O.-P.); jsd@uma.es (J.A.S.-D.); julian@elca.uma.es (J.C.-R.); jahidalgo@uma.es (J.A.H.-L.); 2 Instituto de Investigación Biomédica de Málaga (IBIMA), Málaga 29010, Spain

**Keywords:** resistive sensor arrays, direct sensor-to-digital device interface, FPGAs, parallel analogue data acquisition

## Abstract

One of the most suitable ways of distributing a resistive sensor array for reading is an array with *M* rows and *N* columns. This allows reduced wiring and a certain degree of parallelism in the implementation, although it also introduces crosstalk effects. Several types of circuits can carry out the analogue-digital conversion of this type of sensors. This article focuses on the use of operational amplifiers with capacitive feedback and FPGAs for this task. Specifically, modifications of a previously reported circuit are proposed to reduce the errors due to the non-idealities of the amplifiers and the I/O drivers of the FPGA. Moreover, calibration algorithms are derived from the analysis of the proposed circuitry to reduce the crosstalk error and improve the accuracy. Finally, the performances of the proposals is evaluated experimentally on an array of resistors and for different ranges.

## 1. Introduction

There is a large range of applications which use resistive sensor arrays to obtain information about a specific system, such as temperature sensing [1,2], gas detection [3,4], tactile sensing [5,6,7,8] and others. The complexity of the electronic system necessary to read the information of the array depends on the number of sensors, the number of connections necessary to extract the information, the resistance values of each sensor, and the speed necessary to obtain this information. Moreover, the system is even more complex when subsequent processing, such as in the case of a smart sensor, is required for sending data to a central unit and it is to be done in the circuit which scans the information of the array.

The processing speed of the array signals involves a trade-off with the complexity of the system: if maximum processing speed is required, parallel access to the information of all the individual sensors must be done individually, meaning a high number of wires to carry this information and a large number of processing units in the circuits to receive it.

Indeed, for circuits with maximum parallelism, if the sensors are distributed in a 2 dimensional array with lengths *M* for rows and *N* for columns, the number of wires may reach 2 × *M* × *N*. However, one of the sensor terminals is generally shared, meaning the final number may be reduced to *M* × *N* + 1 [9]. Each of these wires should be connected to a circuit to scan the information of a specific sensor in which the resistance value is translated into a voltage value and subsequently a digital number, meaning *M* × *N* of these circuits would be required. The time to scan the array would approximately coincide with the time to scan an array element.

However, if information extraction speed is not necessarily so high, the number of connections and the complexity of the circuit can be reduced, opting for a connection in rows and columns in which each sensor is connected to a different row and column. In this configuration there are different sensors sharing the rows and columns, but in such a manner that the information of a single sensor can be accessed by selecting a row and column. Here the number of wires is reduced to *M* + *N* and the number of circuits necessary to scan the information would be *M* or *N*, depending on the type of reading of the array. Obviously, additional circuits are required for sensor addressing and, moreover, the time necessary to extract the information is the result of multiplying the time taken to scan a sensor by the number of *N* columns or *M* rows of the array plus the time necessary for multiplexing. This same solution must be used if the construction of the sensor itself has a row and columns structure and its output is provided by *M* + *N* wires [6].

A third solution to access the information of the array with minimum circuitry is to use time multiplexing for the information from the *M* + *N* wires, meaning only one sensor is scanned. An improved version of this type of circuits is the one known as improved isolated drive feedback circuit (IIDFC), proposed in [10]. This solution is slower since it involves multiplying by *M* × *N* the scanning time of a single sensor in order to obtain the information from all the array, to which the time necessary for multiplexing must be added.

In consequence, the designer will opt for one solution or another based on the type of substrate upon which the circuits and the sensors are implemented, the size and architecture of the array, the size and consumption of the scan circuit and the scan time of the array. However, intermediate solutions somewhere between the three presented here may also exist.

In our case, we scan a tactile resistive sensor intended to emulate the skin in applications such as assistive robotics [11]. In systems such as these, the tactile sensor arrays are medium-size (in our case *M* = 8 and *N* = 6) and a scanning frequency of 250 frames per second is sufficient to correctly detect slippage [12]. For this reason, a trade-off between speed and wiring complexity of the system has been chosen, meaning the information contained in all the sensors connected to a single sensor row is accessed simultaneously using six equal circuits (one per column) to obtain a digital scan of the information.

However, any type of multiplexing of the array information implies what in the literature has become known as crosstalk, *i.e.*, the influence of the other sensors on each individual scanning. For this reason the circuitry used should strive to prevent or offset any such phenomena. Specifically, the circuit in [13] exploits active integrators to implement direct resistive sensor-FPGA connection and carry out the analog-to-digital conversion. The negative feedback in the integrator allows also implementing a common strategy to reduce the crosstalk based on grounding [14]. The parasitic resistive paths are short circuited ideally with this approach and crosstalk is cancelled. Unfortunately, non-idealities such as the restricted driving capability of the I/O pins of the FPGA, and their associated impedances, the offset voltage and the bias currents of the operational amplifiers introduce crosstalk effects and limit the performance of this circuit, also limiting the range of resistances that can be measured with a certain accuracy. This paper provides careful analysis to quantify the errors and proposes techniques based on added calibration resistors and proper algorithms to improve the performance of the circuit, achieving larger measuring ranges and higher accuracies.

The article is structured in sections as follows: Section 2 analyses the circuits proposed in literature and selects the most appropriate for our sensor. Section 3 sets out the modifications to the selected circuit, taking into account a series of factors not previously considered (offset voltage of the operational amplifiers, polarization current, measuring errors in elements of the circuit, *etc.*). Section 4 describes the materials used in implementing the circuit. Section 5 shows the experimental results obtained and analyses the consequences of using the equations obtained in Section 3 for interpretation. The final section summarizes the conclusions.

## 2. Readout Circuit with Capacitive Feedback

**Figure 1:**
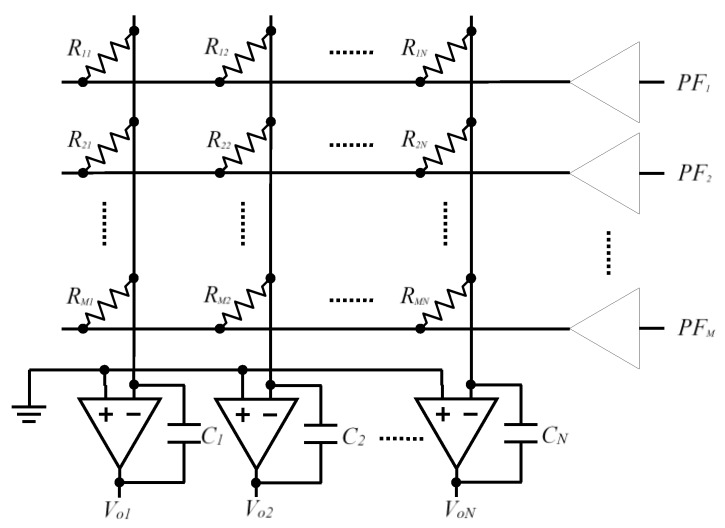
Schematic proposed in [13] for reading of a resistive array.

A circuit with a direct resistive sensor-FPGA interface, without A/D converters, is presented in [13]. This circuit uses operational amplifiers (OAs) with capacitive feedback to implement grounding and reduce crosstalk on the element being tested (hereinafter EBT). This solution is shown in Figure 1.

The resistance value *R_ij_* of an EBT is read by measuring a discharge time through *C_j_*. Initially, the charged capacitor maintains voltage in the OA output node, which is interpreted as a 1-logic in a digital processor input pin. As the capacitor discharges, a voltage is reached which makes the input pin interpret the input as a 0-logic level. The measurement will therefore be the time difference between the capacitor charge finishing and receipt of a 0 through the digital processor input pin. [13] sets out a series of arguments in which the choice of an FPGA is suitable as a processing unit. These include, most notably, that each of the inputs can be processed in parallel by the FPGA programmable hardware.

The way the circuit works is described in more detail below. As illustrated in Figure 1, the sensor row lines are the *M* pin outputs, configured as output of an FPGA. In order to know the resistance of the sensor with value *R_ij_*, the pin of row *i*, *PFi*, is placed at a high level (a value close to *V_DD_* in the FPGA output), and the other row pins at 0-logic value (a value close to 0 V at the FPGA output). This means current will only circulate through the resistors of this row (a single resistor in each column).

**Figure 2:**
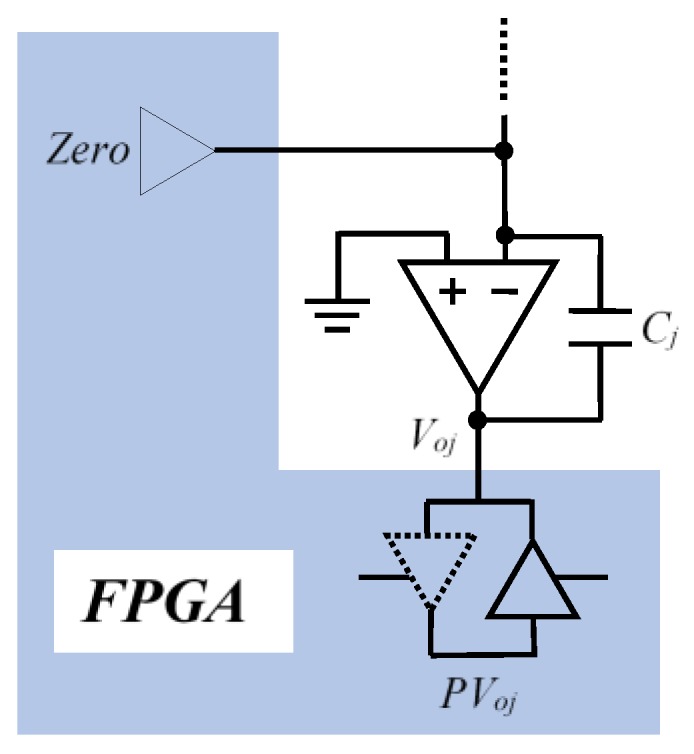
FPGA pins which charge the capacitor in the CHARGE stage.

In order to duly use this current in a first cycle (CHARGE cycle), all the *C_j_* capacitors are charged with *V_DD_* voltage in the OA output terminal, maintaining 0 V in the inverter terminal. This is done via two FPGA output buffers (*Zero* and *PV_oj_* in Figure 2).

It should be noted that the pins which will be in charge of reading *PV_oj_* voltage are configured as output in this phase. The OA will also be disabled (placing the shutdown pin at 0 V) and the FPGA outputs which control the row signals, *PF_k_*, will all be 0 V. A new cycle, ACTIVATE, is then entered, activating the OA and placing shutdown at high level.The pin *PV_oj_* is configured in high impedance at the same time. Finally, the DISCHARGE cycle is carried out, discharging the capacitors. To do this, the *Zero* pin is configured in high impedance and the output buffer of the row to be scanned, *PFi*, is placed at *V_DD_*, with all other row buffers remaining at 0 V. At the same time, the OA output voltage reading pins, *PV_oj_*, are configured as input. This means the output voltage of all OAs will decrease as the current which crosses the different *R_ij_* discharges the capacitors.

Operating in this manner, all *PV_oj_* pins will have an input of value *V_DD_* volts at the start of the DISCHARGE cycle, which will decrease as time passes through to a value *VT_j_*, the threshold voltage for which the *PV_oj_* pin input buffers start to interpret the input as a 0-logic which is transmitted to inside the FPGA. The time in which *V_oj_* passes from *V_DD_* to *VT_j_* in the DISCHARGE cycle will be known as Δ*t_ij_*. Considering the OA ideal and the resistance value of the sensor constant, a simple analysis would indicate that:

(1) $$\Delta t_{ij}=R_{ij}C_{j}\frac{V_{DD}-VT_{j}}{V_{DD}}$$

in which there is a linear relationship between time measured Δ*t_ij_* and resistance value *R_ij_*.

The main advantages of the circuit proposed in [13] are the elimination of the AD converter and the possibility of parallel processing in the FPGA of the information from the series of sensors which are scanned simultaneously. This configuration therefore allows greater processing speed and a reduced area and consumption.

An additional row, calibration row, is added in [13], in order to not have to evaluate *C_j_* and *VT_j_* in Equation (1), since these magnitudes are difficult to measure due to their value varying with the supply voltage, time and temperature. This procedure is equivalent to what is known in literature as single-point calibration [15]. However, this type of calibration does not take into account the resistances of the buffers of each row, *k*, of the FPGA, *RB_k_*, which results in an error in estimating *R_ij_*. If the *RB_k_* values were constant and equal for all buffers, the value could be calculated with a Two-Points calibration [15], which would involve adding a second calibration row to that proposed in [13]. Moreover, *RB_k_* although it is constant in a DISCHARGE cycle, it is not equal in all buffers, since it depends on the array resistors it is connected to, meaning even adding a second calibration row would not avoid the errors due to this resistor. It is important to note that we have to evaluate its value and also avoid the effect of crosstalk which comes about from being joined to several array resistors, one resistor per column, a question which a two-point calibration does not resolve. Furthermore, it should be remembered that the OAs used are not ideal and, in consequence, present second-order effects, which also results in crosstalk, as will be shown in more detail below. Finally, [13] does not include any analysis of the possible ranges of resistance values which the circuit may measure correctly.

## 3. Improving Circuits with Capacitive Feedback for Readout Resistive Sensor Arrays

Each paragraph in this section analyses and proposes solutions for each of the problems set out above.

### 3.1. Estimation of R_ij_ Considering the Effect of the Resistance of the Row Selection Buffers and Variations in the Values C_j_ and VT_j_

**Figure 3:**
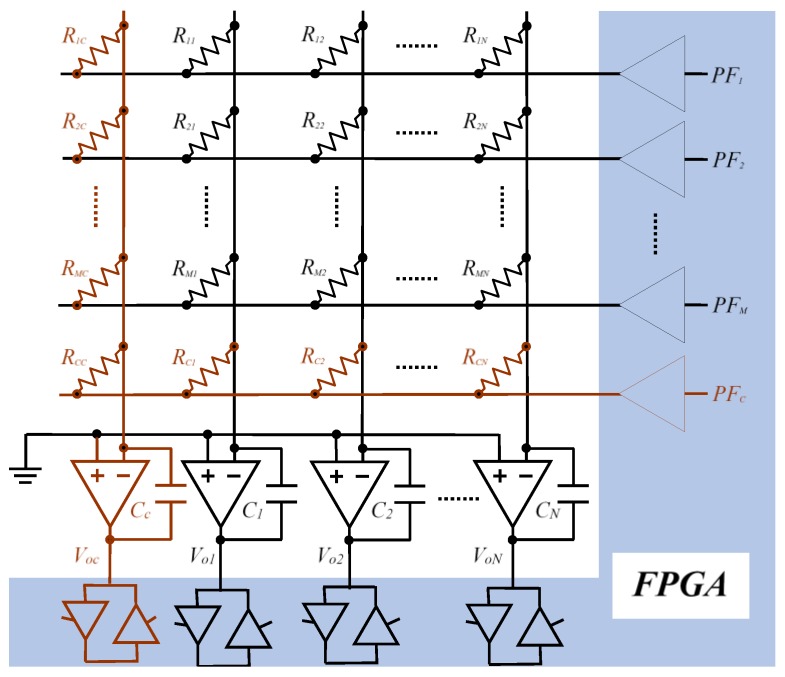
Simplified schematic of the modification proposed for the circuit of Figure 1, in order to consider the buffer resistances and avoid measurement errors in *C_j_* and *VT_j_*.

In order to reduce the effects of variation in measurements *C_j_* and *VT_j_* and to simultaneously eliminate the effect of the resistance of the row selection buffers, using the circuit in Figure 3 is proposed. The so-called calibration row and column have been added to it (in red). The circuit therefore has *M* + 1 rows and *N* + 1 columns. In consequence, *N* + *M* + 1 additional resistors with known values need to be used, along with an extra OA in the calibration column.

**Figure 4:**
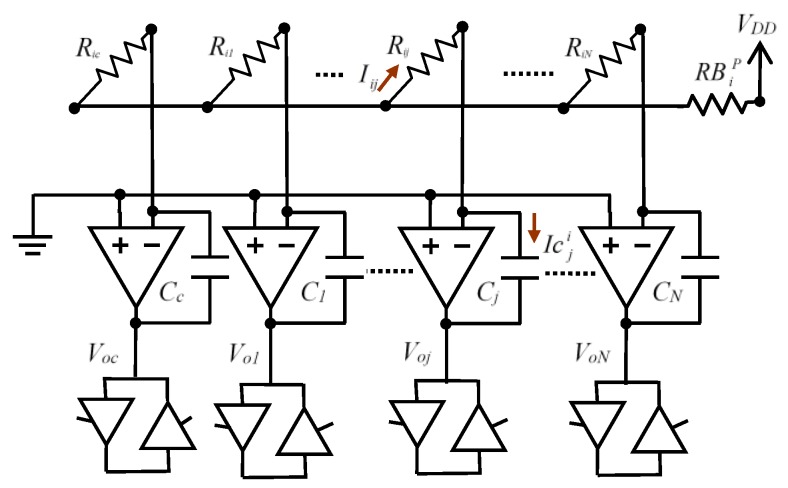
Equivalent circuit to scan the values of the resistors of row *i*.

The operation of the circuit is exactly the same as indicated in the paragraph above, taking into account that there is now one more row to read (for simplicity, the part marked in blue in Figure 2 is not shown). Figure 4 shows the equivalent circuit to scan the resistors of row *i*. Here the row control buffer has been replaced with the corresponding output resistor *RB_k_*. In these buffers the resistors for high status output, $RB_{k}^{p}$, are different to those shown in a low status output, $RB_{k}^{n}$, although both can be small (10–50 Ω), due to the CMOS technology used in manufacture.

Supposing that the OA are ideal, the value for Δ*t_ij_* would be given in the Equation (1), although this does not take into account the different *RB_k_*. To illustrate the influence of these resistors, the current, $Ic_{j}^{i}$, which enters through resistor *R_ij_* and discharges capacitor *C_j_* when row *i* is activated, is calculated. If $RP_{i}=R_{i1}\|R_{i2}\|\;...\;\|R_{iN}\|R_{ic}$ is the parallel of all resistors being scanned, including the calibration resistor, a simple analysis of the circuit of Figure 4 shows:

(2) $$Ic_{j}^{i}=I_{Rij}=\frac{V_{DD}}{R_{ij}}\cdot \frac{RP_{i}}{RB_{i}^{p}+RP_{i}}$$

meaning the time to discharge *C_j_* at *VT_j_* is given by:

(3) $$\Delta t_{ij}=C_{j}\frac{V_{DD}-VT_{j}}{I_{ij}}=R_{ij}C_{j}\frac{V_{DD}-VT_{j}}{V_{DD}}\cdot \frac{RB_{i}^{p}+RP_{i}}{RP_{i}}$$

It is worthwhile noting that the Equation (3) shows the appearance of crosstalk in the circuit, since Δ*t_ij_* is no longer a function of a single resistor of the array, *R_ij_*, but rather of all resistors of the same row (through *RP_i_*). Hence, Δ*t_ij_* should be calculated by way of a non-linear system of *N* + 1 equations in which it is necessary to know the exact values of *C_j_*, *VT_j_*, $RB_{i}^{p}$ and the discharge times for all the columns when row *i* is activated. It is also necessary to take into account that $RB_{i}^{p}$ is not constant and varies throughout the discharge process. The details below indicate how to proceed in order to avoid these inconveniences in the *R_ij_* calculation using different array measurement times.

Once the scanning process for all resistors has been completed, these times are stored in the FPGA. Hence, Δ*t_ic_* can be used to calculate the Δ*t_ij_*/Δ*t_ic_* coefficient and express *R_ij_* based on the Equation (3) as follows:

(4) $$R_{ij}=\left(\frac{C_{c}}{C_{j}}\cdot \frac{V_{DD}-VT_{c}}{V_{DD}-VT_{j}}\right)\cdot \frac{\Delta t_{ij}}{\Delta t_{ic}}R_{ic}$$

In this expression only the part between parentheses is unknown; however, as there is also Δ*t_cj_* and Δ*t_cc_* its value can be found by proceeding in the same way as when finding the expression (4):

(5) $$\frac{C_{c}}{C_{j}}\cdot \frac{V_{DD}-VT_{c}}{V_{DD}-VT_{j}}=\frac{\Delta t_{cc}}{\Delta t_{cj}}\frac{R_{cj}}{R_{cc}}$$

replacing Equation (5) in Equation (4):

(6) $$R_{ij}=R_{ic}\frac{R_{cj}}{R_{cc}}\frac{\Delta t_{ij}}{\Delta t_{ic}}\frac{\Delta t_{cc}}{\Delta t_{cj}}$$

In the term on the right of Equation (6), once the sensor array has been scanned, all the terms are known and, in consequence, it is not necessary to modify the scanning process indicated in Section 2 in order to obtain the values of the different *R_ij_* resistors. The operations necessary to find *R_ij_* can be carried out in the FPGA and their results transmitted or processed whilst extracting data from a new sensor array frame.

The Equation (6) can also be used in line with the digital number of cycles (*D*) which the FPGA uses to measure the different times of this equation. Hence, if Δ*t* = *D* × *T_s_*, where *T_s_* is the meter clock period, Equation (6) the result is:

(7) $$R_{ij}=R_{ic}\frac{R_{cj}}{R_{cc}}\frac{D_{ij}}{D_{ic}}\frac{D_{cc}}{D_{cj}}$$

It should be noted that in Equation (2), in order to deduce *R_ij_*, a series of resistors *RB_k_* have been used which model the operation of each buffer; however, if *R_ij_* is evaluated by way of Equation (6), these effects do not have any influence since *RB_k_* does not appear in the expression.

### 3.2. Limitations in the Range of Resistors to Be Measured

In order to use the Equation (6) correctly, it is necessary to determine the range of resistors which can be measured with the circuit of Figure 3. Two main circumstances limit this range. Firstly, the maximum current which the FPGA can provide will depend on each specific model and must be carefully examined in the design stage. For the correct operation of the circuit, it does not matter that the buffer output provides a voltage below *V_DD_* in a specific amount, since the limitation actually comes from the maximum current which can be provided by a specific buffer or series of buffers which select the rows of the array without affecting the correct operation of the FPGA.

Secondly, it should be noted that the current provided by each buffer will depend inversely on *RP_i_*, and, in consequence, will increase with the number of array columns or with the reduction of the resistance values. This limits the size of the array to be scanned and the possible array resistor ranges. In order to prevent this restriction, the resistor of each row to be scanned can be increased by adding an *RS_i_* series resistor at the output of each buffer, which will be added to *RB_k_*, thus reducing the current provided by the buffer. Although this resistor is added, the Equation (6) can continue to be used to calculate *R_ij_*.

However, there is another limitation related to the range of resistors to be measured, since each resistor of the sensors of a row takes different times to discharge the capacitor of its column. Hence, whilst row *i* has the lowest possible value resistor, *RL* (in column *l*), and another with the highest possible value, *RH* (in column *h*), it may occur that the former completely discharges its capacitor to 0 V whilst the latter has not yet achieved output voltage below *VT_h_*. In this situation, the capacitor of column *l* continues to receive current from the sensor resistor, but, as *V_ol_* has reached its minimum value, the OA enters non-linear operation mode (even when it is a rail-to-rail OA), meaning the voltage in the OA inverter input node starts to increase, and ceases to be a virtual ground. If this occurs, the crosstalk phenomenon will appear through the resistors of the non-selected rows (as shown in Section 3.4), affecting the resistor time measurement *RH* which is as yet incomplete.

*I_RH_* and *I_RL_* are the currents which cross the *RH* and *RL* resistors respectively. The relation which must be met in order to prevent the aforementioned situation from coming about is set out below.

The output voltage of a column in DISCHARGE phase is given by:

(8) $$V_{o}=V_{DD}-\frac{I\cdot t}{C}$$

Hence, the time necessary for *RL* to discharge the capacitor of its column to 0 V will be:

(9) $$t_{RL}=\frac{V_{DD}\cdot C_{L}}{I_{RL}}$$

In order for crosstalk effect to be prevented, in this time the output voltage of column *h* must have dropped below the threshold value *VT* (for the purpose of simplicity, it is considered that the threshold voltages of all rows are equal). Hence, in accordance with Equation (8):

(10) $$V_{oh}(t=t_{RL})=V_{DD}-\frac{I_{RH}\cdot t_{RL}}{C_{H}}<VT$$

replacing Equation (9) in Equation (10) and supposing, for the purpose of simplicity, the capacitors of all the columns are equal, we obtain:

(11) $$\frac{I_{RL}}{I_{RH}}<\frac{V_{DD}}{V_{DD}-VT}$$

The Equation (2) can be used to find the values for *I_RL_* and *I_RH_*, and replacing them in Equation (11) we obtain:

(12) $$RH<\frac{V_{DD}}{V_{DD}-VT}\cdot RL$$

Hence, Equation (12) shows the limitations in the possible sensor resistance values in order for the circuit to work correctly. In the implementation carried out in Section 4, using a Spartan 3 XCS50AN-4TQG144C, with *V_DD_* = 3.3 V results in *VT* = 1.4 V, meaning *RH* < 1.74 × *RL*.

As will also be shown in Section 4, for *RL* values over, approximately, 3 kΩ, the effects of failing to meet Equation (12) are very small. Moreover, it is possible to prevent increased voltage of the OA inverter node by activating the *Zero* pin of the FPGA at the moment the output node reaches voltage *VT*. However, for lower *RL* values of the array, crosstalk effect is increasingly important and Equation (12) is a serious limitation in the circuit.

### 3.3. Increasing the Range of Resistors

In order to increase the range of resistors to be measured, it is not necessary to modify the design of the Figure 3 but only to carry out the reading of two simultaneous rows: the sensors array, *i*, and the calibration resistors row, *c*.

Doing this, there are two resistors through which the capacitor of each column is discharged. If the maximum and minimum resistors of the array, *RH* and *RL*, are found again in the row to be scanned, in columns *h* and *l* simultaneously, the calibration resistors of the same columns are scanned: *R_C_* (all with the same value), so the Equation (11) is now transformed into:

(13) $$\frac{I_{RL}+I_{R_{c}}}{I_{RH}+I_{Rc}}<\frac{V_{DD}}{V_{DD}-VT}$$

Replacing the values of the currents obtained using Equation (2) in this expression, the following can be written:

(14) $$RH<\frac{V_{DD}}{V_{DD}-VT}\cdot RL+\frac{VT}{V_{DD}-VT}\cdot \frac{RP_{c}}{RP_{i}}\cdot \frac{RB_{i}^{p}+RP_{i}}{RB_{c}^{p}+RP_{c}}\cdot \frac{RH\cdot RL}{R_{C}}$$

in which the threshold voltages and the capacitors of all the rows have again be taken as equal. Regrouping all the terms with *RH* in the left member of Equation (14), the following can be written:

(15) $$RH\cdot \left[V_{DD}-VT\cdot \left(1+\frac{RP_{c}}{RP_{i}}\cdot \frac{RB_{i}^{p}+RP_{i}}{RB_{c}^{p}+RP_{c}}\cdot \frac{RL}{R_{C}}\right)\right]<V_{DD}\cdot RL$$

A simpler expression can be achieved with the approximation $RB^{p}<<RP$ (the resistors of the row selection buffers have very low values), meaning Equation (15) obtains an upper limit for *RH* as:

(16) $$RH<\frac{V_{DD}\cdot RL}{V_{DD}-VT\cdot \left(1+\frac{RL}{R_{C}}\right)}$$

Comparing the Equations (12) and (16) shows how the range has been extended. The restrictions on *RH* can also be eliminated by making the denominator of Equation (16) equal to 0. This means that *R_C_* should be:

(17) $$R_{C}=\frac{VT}{V_{DD}-VT}\cdot RL$$

In the previous design example *R_C_* = 0.74 × *RL*.

However, if the restriction $RB^{p}<<RP$ is not met, either because the number of columns is large, the *RL* values are small, or a series resistor has been introduced with the buffer, then *RH* will continue to be limited by Equation (15); however, if in this expression the term between square brackets is below or equal to 0, any *RH* value would be possible. Modifying Equation (15), this condition can be written as:

(18) VDD−VT⋅(1+RBipRPi+1RBcp(N+1)+RC⋅RL)≤0

In order to meet the restriction, the *R_C_* and $RB_{i}^{p}$ values can be modified (including a series resistor with the buffer, *RS_i_*). Hence, a higher limit can be found for the *R_C_* value and a lower limit for $RB_{i}^{p}+RS_{i}$. These limits will be more restrictive when *RP_i_* is maximum, *RP_imax_* found in accordance with the following expression:

(19) $$\frac{1}{RP_{i\max}}=\frac{1}{RL}+\frac{1}{R_{ic}}+\frac{N-1}{RH}$$

Taking into account all the foregoing, *R_C_* can be cleared in Equation (18) obtaining:

(20) $$R_{C}<\frac{VT}{V_{DD}-VT}\cdot RL\cdot \left(1+\frac{RB_{i}^{p}+RS_{i}}{RP_{i\max}}\right)-RB_{c}^{p}(N+1)$$

It is clear that the member of the right of Equation (20) must be greater than 0, but this can always be achieved thanks to *RS_i_*, even when the number of columns *N* is large. However, in order to obtain Equation (20), two rows of the array are selected simultaneously, and, in consequence, the expression Equation (6) is no longer valid since it is not a single current which discharges the capacitor but rather the sum of two: the current which circulates through resistor *R_ij_* and the one which circulates through resistor *R_cj_*.

In order to find an expression equivalent to Equation (6) and to determine the value of *R_ij_*, 4 discharge times are used: Δ*t′_ij_*, Δ*t′_ic_*, Δ*t_cj_* and Δ*t_cc_*. The first two are the discharge times of the columns *j* and *c* when rows *i* and calibration row *c* are selected simultaneously. Δ*t_cj_* and Δ*t_cc_* are the discharge times of columns *j* and *c* when only the calibration row is selected. The expression Equation (3) can be used to calculate Δ*t_cj_* and Δ*t_cc_*, whilst to calculate Δ*t′_ij_*, Δ*t′_ic_* only two currents discharge the capacitor and in consequence:

(21) $$\begin{array}{l} \Delta t_{ij}^{\prime }=C_{j}\frac{V_{DD}-VT_{j}}{I_{ij}+I_{cj}}\\ \Delta t_{ic}^{\prime }=C_{c}\frac{V_{DD}-VT_{c}}{I_{ic}+I_{cc}} \end{array}$$

Taking into account these expressions and using those obtained in Equation (2) for the currents, *R_ij_* can be expressed as:

(22) $$R_{ij}=\frac{R_{cj}R_{ic}}{R_{cc}}\frac{\Delta t_{ij}^{\prime }}{\Delta t_{ic}^{\prime }}\frac{\Delta t_{cj}}{\Delta t_{cc}}\left(\frac{\Delta t_{cc}-\Delta t_{ic}^{\prime }}{\Delta t_{cj}-\Delta t_{ij}^{\prime }}\right)$$

Hence, modifying the array scanning procedure and selecting the appropriate values of *R_C_* means very wide ranges of resistors can be measured, and, knowing four discharge times, the value of *R_ij_* determined. It should be highlighted that the time spent scanning the whole array in order to obtain the data of Equation (6) is the same as the time taken to obtain the data of Equation (22), and that the number of time measurements to be saved in the internal memory of the FPGA is the same. Moreover, the calculations to obtain *R_ij_* by Equation (22) can be carried out in the FPGA whilst data are obtained for a new array frame.

### 3.4. Crosstalk Due to the Offset Voltages of the Operational Amplifiers

The OAs present offset voltages, *θ*, which, depending on the models used, can vary in the range between millivolts and microvolts. As the offset voltages are random values determined by variations in the transistor manufacture processes, each OA (even when it is the same model) can have a different value. Moreover, as the OA non-inverter input voltage is set to ground in the circuit, the offset voltage appears in the OA inverter terminal. For this reason, the inverter input voltages of all OAs can vary, resulting in the appearance of crosstalk.

**Figure 5:**
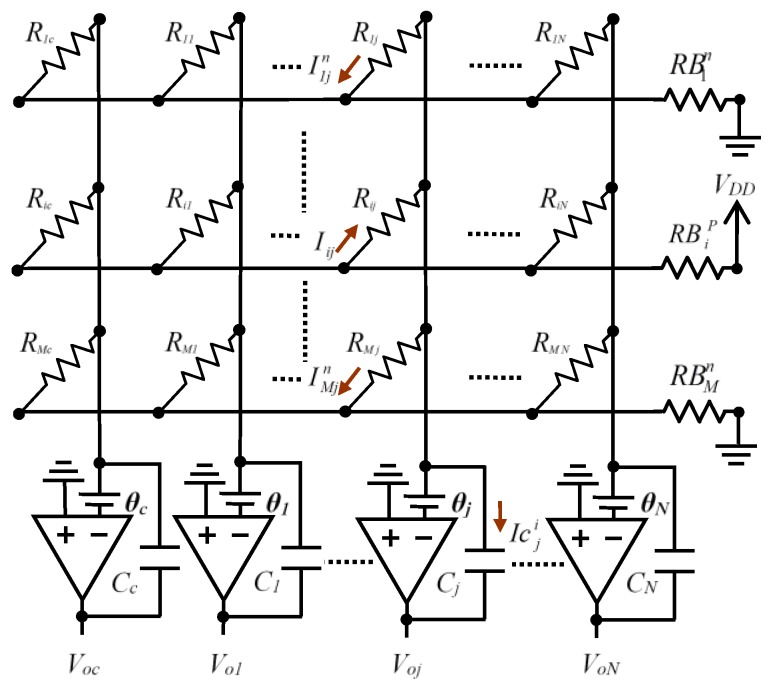
Equivalent circuit for the activation of a single row.

The circuit used to analyse crosstalk due to the offset of the OAs is indicated in Figure 5, which shows the different offset voltages of the OAs, *θ_j_*; j∈{1,2,...N,c} and the situation in which a single row of sensors, row *i*, is scanned is illustrated. All FPGA row control buffers have been replaced with their corresponding resistors, $RB_{i}^{p}$, for the case of rows selected at *V_DD_* or $RB_{i}^{n}$ for rows at 0 V.

**Figure 6:**
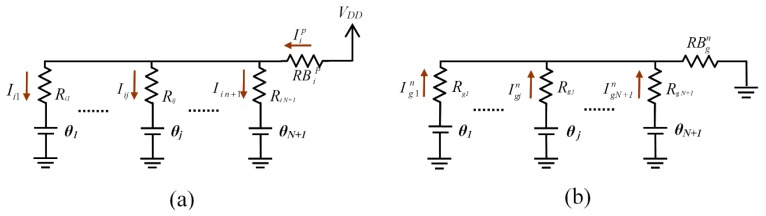
Detail of the circuit for calculation of currents during activation of row *i*. (**a**) Currents for the selected row *i*. (**b**) Currents for a non-selected row *g*.

The value to be found is the current $Ic_{j}^{i}$ which enters capacitor *C_j_*. This current has several components. Figure 6 can be used for analysis, showing in detail the components of $Ic_{j}^{i}$.

The first component, *I_ij_*, which comes from the source *V_DD_* through $RB_{i}^{p}$ and crosses *R_ij_*, can be analysed by simply observing that all the row resistors are connected at one end to $RB_{i}^{p}$ and at the other end to a source of value *θ_j_*. Moreover, if $I_{i}^{p}$ is defined as the current which flows through $RB_{i}^{p}$, then it is obtained by:

(23) $$I_{i}^{p}=\sum_{k=1}^{N+1}I_{ik}$$

Observing Figure 6a, all the currents which flow through the resistors of row *i* can be set out in accordance with *I_ij_* since:

(24) $$I_{ij}\cdot R_{ij}+\theta_{j}=I_{ik}\cdot R_{ik}+\theta_{k}\;\forall k\in \left\{1,2,...N,c\right\}$$

Hence, using this equation, we can write:

(25) Iip=∑k=1N+1Iik=Iij⋅RijRPi+∑k=1N+1θjkRik

where *θ_jk_* = *θ_j_* − *θ_k_* and *k* = *N* + 1 is the calibration column.

Taking into account that *V_DD_* can be expressed:

(26) $$V_{DD}=I_{i}^{p}\cdot RB_{i}^{p}+I_{ij}\cdot R_{ij}+\theta_{j}$$

*I_ij_* can be cleared using Equation (25):

(27) $$I_{ij}=\left(V_{DD}-\theta_{j}-RB_{i}^{p}\cdot \sum_{k=1}^{N+1}\frac{\theta_{jk}}{R_{ik}}\right)\cdot \frac{RP_{i}}{RB_{i}^{p}+RP_{i}}\cdot \frac{1}{R_{ij}}$$

Secondly there are all the currents which, through the rest of the resistors of column *j*, are drained to ground, Figure 6b. As shown in this figure, $I_{gj}^{n}$ is the current which flows through the resistor, *R_gj_*, with g ≠ i. Using the same analysis as for the calculation of *I_ij_* in Figure 6a (replacing *V_DD_* with 0 V, $RB_{i}^{p}$ with $RB_{i}^{n}$ and taking the appropriate indices) we arrive at:

(28) $$I_{gj}^{n}=\left(\theta_{j}+RB_{g}^{n}\cdot \sum_{k=1}^{N+1}\frac{\theta_{jk}}{R_{gk}}\right)\cdot \frac{RP_{g}}{RB_{g}^{n}+RP_{g}}\cdot \frac{1}{R_{gj}}$$

As current is drained to ground through all the resistors of column *j* with the exception of *R_ij_*, this can be calculated as:

(29) $$Ic_{j}^{i}=I_{ij}-\sum_{g=1}^{M+1}I_{gj}^{n}+I_{ij}^{n}$$

where *g* = *M* + 1 is the calibration row. Replacing Equation (27) and Equation (28) in Equation (29) one obtains:

(30) $$\begin{array}{c} Ic_{j}^{i}=\frac{V_{DD}}{RB_{i}^{p}+RP_{i}}\cdot \frac{RP_{i}}{R_{ij}}+\frac{RP_{i}\left(RB_{i}^{n}-RB_{i}^{p}\right)}{\left(RB_{i}^{n}+RP_{i}\right)\left(RB_{i}^{p}+RP_{i}\right)}\cdot \frac{RP_{i}}{R_{ij}}\cdot \sum_{k=1}^{N+1}\frac{\theta_{jk}}{R_{ik}}+\\ +\theta_{j}\cdot \frac{RB_{i}^{p}-RB_{i}^{n}}{\left(RB_{i}^{n}+RP_{i}\right)\left(RB_{i}^{p}+RP_{i}\right)}\cdot \frac{RP_{i}}{R_{ij}}-\theta_{j}\sum_{g=1}^{M+1}\frac{RP_{g}}{RB_{g}^{n}+RP_{g}}\cdot \frac{1}{R_{gj}}-\\ -\sum_{g=1}^{M+1}\frac{RB_{g}^{n}\cdot RP_{g}}{RB_{g}^{n}+RP_{g}}\cdot \frac{1}{R_{gj}}\sum_{k=1}^{N+1}\frac{\theta_{jk}}{R_{gk}} \end{array}$$

where, separating *θ_jk_* = *θ_j_* − *θ_k_*, we can write:

(31) $$\begin{array}{c} Ic_{j}^{i}=\frac{V_{DD}}{RB_{i}^{p}+RP_{i}}\cdot \frac{RP_{i}}{R_{ij}}+\frac{RP_{i}\left(RB_{i}^{p}-RB_{i}^{n}\right)}{\left(RB_{i}^{n}+RP_{i}\right)\left(RB_{i}^{p}+RP_{i}\right)}\cdot \frac{RP_{i}}{R_{ij}}\cdot \sum_{k=1}^{N+1}\frac{\theta_{k}}{R_{ik}}+\\ -\theta_{j}\sum_{g=1}^{M+1}\frac{1}{R_{gj}}+\sum_{g=1}^{M+1}\frac{RB_{g}^{n}}{RB_{g}^{n}+RP_{g}}\cdot \frac{RP_{g}}{R_{gj}}\sum_{k=1}^{N+1}\frac{\theta_{k}}{R_{gk}} \end{array}$$

an expression which characterises the crosstalk of the circuit when the inverter input of the OAs does not have a virtual ground voltage.

It should be noted that this expression can be written in abbreviated form:

(32) $$Ic_{j}^{i}=\frac{F(i)}{R_{ij}}+G(j)$$

where *F*(*i*) is a function which depends on index *i* but not on *j*:

(33) $$F(i)=\frac{V_{DD}\cdot RP_{i}}{RB_{i}^{p}+RP_{i}}+\frac{RP_{i}^{2}\left(RB_{i}^{p}-RB_{i}^{n}\right)}{\left(RB_{i}^{n}+RP_{i}\right)\left(RB_{i}^{p}+RP_{i}\right)}\cdot \sum_{k=1}^{N+1}\frac{\theta_{k}}{R_{ik}}$$

and *G*(*j*) is a function which depends on index *j* but not on *i*:

(34) $$G(j)=-\;\theta_{j}\sum_{g=1}^{M+1}\frac{1}{R_{gj}}+\sum_{g=1}^{M+1}\frac{RB_{g}^{n}\cdot RP_{g}}{RB_{g}^{n}+RP_{g}}\cdot \frac{1}{R_{gj}}\sum_{k=1}^{N+1}\frac{\theta_{k}}{R_{gk}}$$

Moreover, using Equation (28) it is easy to check that:

(35) $$\sum_{g=1}^{M+1}I_{gj}^{n}=-\;G(j)$$

a result which will be used in the following section.

In this manner, dependence on $Ic_{j}^{i}$ is separated in: one term for rows and another for columns and the value of *R_ij_*. This will allow us to, as will be seen in the following section, design a strategy to find the value of *R_ij_* taking into account the effects of the offset voltages of the OA. It can also be seen how from the Equation (31), with all offset voltages at 0, we can derive the Equation (2).

Returning to the Equation (31), the operation of the crosstalk in the array can be analysed. Hence, if the resistors of buffers $RB^{n}$ and $RB^{p}$ are very small, or small compared to *RP*, Equation (31) this is simplified:

(36) $$Ic_{j}=\frac{V_{DD}}{RB_{i}^{p}+RP_{i}}\cdot \frac{RP_{i}}{R_{ij}}-\theta_{j}\sum_{g=1}^{M+1}\frac{1}{R_{gj}}$$

where the crosstalk effect due to resistors of other columns has disappeared. Hence, $Ic_{j}^{i}$ is only modified, with regard to the Equation (2), by the resistors of the same column, *j*, and by *θ_j_*.

This may not be the case if an extension were necessary in the range of resistor values, since *RS_i_* may have to be added to $RB_{i}^{p}$ if the Equation (20) so requires. However, this provides a design guide, since *RS_i_* must be as small as possible in order to reduce the crosstalk.

Equation (31) also shows how an increase in the minimum values of the *R_ij_* resistors of the array brings a larger decrease in terms 2 and 4 of the right side of the equation with regards to the first (since these have a quadratic dependence with the array resistors) meaning, if the resistors are not large enough, these terms could be eliminated and Equation (31) would be again reduced to Equation (36).

Moreover, an increase in the number of rows or columns due to the summations which appear in Equation (31) means an increase in crosstalk through terms 2, 3 and 4 of the right member of this equation. In consequence, even with high minimum array resistor values, if this has a large number of rows and columns, the crosstalk effect may be the factor which most influences the current which crosses a resistor, even more than the value of the resistor itself.

### 3.5. R_ij_ Calculation Taking into Account the Offset Voltages of the Operational Amplifiers

**Figure 7:**
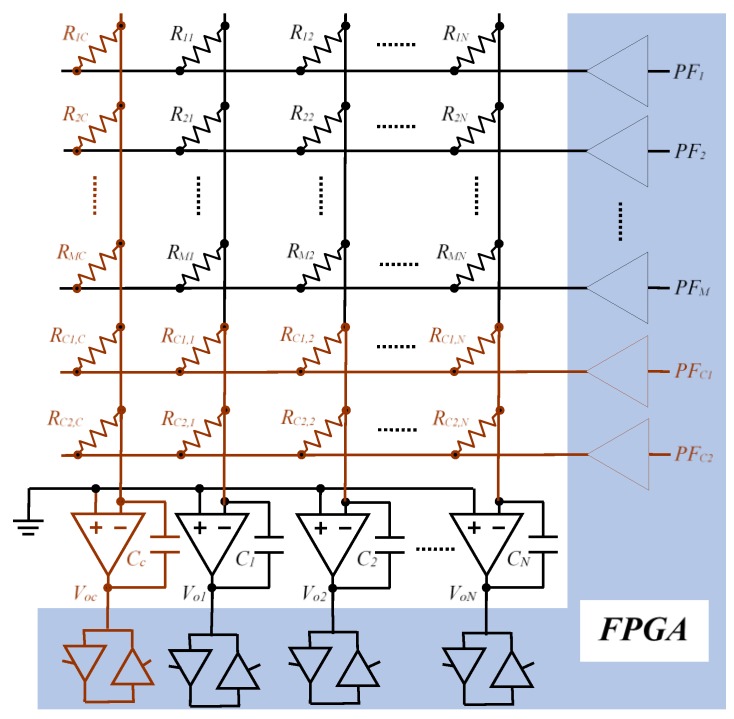
Proposed modification to evaluate the effect of the offset voltages and polarization currents of the OAs.

This section sets out a simple method to obtain the *R_ij_*, values taking into account all the second-order effects presented so far. To apply this method, it is necessary to modify the circuit of Figure 3, adding a second calibration row (these rows will be called *c1* and *c2*), as shown in Figure 7.

Again the method is based on modifying the row reading procedure and the use of different discharge times in order to, firstly, eliminate the term *G*(*j*) from the Equation (32) and, secondly, use simple coefficients to find the resistor values, eliminating *F*(*i*). The process requires the following steps:

- *Step-1*: A row, *i*, of the array and row *c1* are activated simultaneously. The process is repeated for each of the array rows. A series of *M* times Δ*t′_ij_*, the times taken to discharge the different capacitors of the columns when rows *i and c1* are activated simultaneously, are therefore obtained.
- *Step-2*: Row *c1* is activated, obtaining the times Δ*t_c1,j_*.
- *Step-3*: Row *c1* and *c2* are activated simultaneously, obtaining times Δ*t′_c2,j_*.

It should be noted that Steps 2 and 3 are only carried out once during the scanning of all the array rows, and that the three steps can be carried out in any order. The process to obtain *R_ij_* based on the previous steps is shown below.

Following the same procedure as used to find Equation (29), in *Step-1* we would have:

(37) $$Ic_{j}^{i,c1}=I_{ij}+I_{c1j}-\sum_{g=1}^{M+1}I_{gj}^{n}+I_{ij}^{n}+I_{c1j}^{n}=\frac{C_{j}\left(V_{DD}-VT_{j}\right)}{\Delta t_{ij}^{\prime }}$$

where $Ic_{j}^{i,c1}$ indicates the current which discharges capacitor *C_j_* simultaneously activating rows *i* and *c1*. Subtracting the current, $Ic_{j}^{i}$, found during *Step-2:*

(38) $$Ic_{j}^{c1}=I_{c1j}-\sum_{g=1}^{M+1}I_{gj}^{n}+I_{c1j}^{n}=\frac{C_{j}\left(V_{DD}-VT_{j}\right)}{\Delta t_{c1,j}}$$

we obtain:

(39) $$Ic_{j}^{i,c1}-Ic_{j}^{c1}=I_{ij}+I_{ij}^{n}=C_{j}\left(V_{DD}-VT_{j}\right)\left(\frac{1}{\Delta t_{ij}^{\prime }}-\frac{1}{\Delta t_{c1,j}}\right)$$

Taking into account Equation (29), Equation (32) and Equation (35) we can write:

(40) $$I_{ij}+I_{ij}^{n}=\frac{F(i)}{R_{ij}}=C_{j}\left(V_{DD}-VT_{j}\right)\left(\frac{1}{\Delta t_{ij}^{\prime }}-\frac{1}{\Delta t_{c1,j}}\right)$$

likewise, for column *c*:

(41) $$Ic_{c}^{i,c1}-Ic_{c}^{c1}=I_{ic}+I_{ic}^{n}=\frac{F(i)}{R_{ic}}=C_{c}\left(V_{DD}-VT_{c}\right)\left(\frac{1}{\Delta t_{ic}^{\prime }}-\frac{1}{\Delta t_{c1,c}}\right)$$

The process is repeated with the currents of *Step-3*, $Ic_{j}^{c2,c1}$, and *Step-2*, $Ic_{j}^{c1}$, obtaining two equations similar to Equations (40) and (41):

(42) $$I_{c2j}+I_{c2j}^{n}=\frac{F(c2)}{R_{c2j}}=C_{j}\left(V_{DD}-VT_{j}\right)\left(\frac{1}{\Delta t_{c2j}^{\prime }}-\frac{1}{\Delta t_{c1,j}}\right)$$

(43) $$I_{c2c}+I_{c2c}^{n}=\frac{F(c2)}{R_{c2c}}=C_{c}\left(V_{DD}-VT_{c}\right)\left(\frac{1}{\Delta t_{c2c}^{\prime }}-\frac{1}{\Delta t_{c1,c}}\right)$$

The members of the right of Equations (40) and (42) are now divided by the members of the right of Equations (41) and (43), proceeding in the same way for the members of the left. Operating with these ratios finally:

(44) $$R_{ij}=\frac{R_{ic}R_{c2j}}{R_{c2c}}\cdot \frac{\Delta t_{ij}^{\prime }}{\Delta t_{ic}^{\prime }}\cdot \frac{\Delta t_{c2c}^{\prime }}{\Delta t_{c2j}^{\prime }}\cdot \frac{\Delta t_{c1,c}-\Delta t_{ic}^{\prime }}{\Delta t_{c1,j}-\Delta t_{ij}^{\prime }}\cdot \frac{\Delta t_{c1,j}-\Delta t_{c2j}^{\prime }}{\Delta t_{c1,c}-\Delta t_{c2c}^{\prime }}$$

As resistances *R_ic_*, *R_c2j_* and *R_c2c_* and the times are known, the value of *R_ij_* can be calculated again without taking into account the value of the capacitors, *VT* or buffer resistors, *RB*. It should also be noted that the fact that rows *i* and *c1* can be activated simultaneously allows the extension of the array resistor ranges as seen in Section 3.3.

It should be noted that, although the offset voltage has been compensated using the Equation (44), it would be necessary to add to the terms *θ_j_* a term *V_oj_*/*A* due to the finite gain of the OA. However, as will be seen in Section 4, this term takes a much lower value than offset voltage, for which reason it is not taken into account.

### 3.6. Elimination of Effects of the Polarization Currents of the Operational Amplifiers

The Equation (44) also take into account the effects of the polarization current which would enter the OA through the inverter terminal, *Ib_j_*. In effect, if *Ib_j_* is taken into account, Equations (37) and (38) must be modified:

(45) $$Ic_{j}^{i,c1}=I_{ij}+I_{c1j}-\sum_{g=1}^{M+1}I_{gj}^{n}+I_{ij}^{n}+I_{c1j}^{n}-Ib_{j}$$

(46) $$Ic_{j}^{c1}=I_{c1j}-\sum_{g=1}^{M+1}I_{gj}^{n}+I_{c1j}^{n}-Ib_{j}$$

As it appears in the same way in both, it disappears when obtaining the subtraction $Ic_{j}^{i,c1}-Ic_{j}^{c1}$, as is also the case with the three current subtractions carried out to obtain Equation (44), meaning this equation continues to be valid, even considering the polarization currents.

## 4. Materials and Methods

The circuits proposed have been carried out on an FPGA Spartan3AN by Xilinx (XC3S50AN-4TQG144C) [16] with a working frequency of 50 MHz. The meter used by the capture modules is 14 bits, with a base time of 20 ns. The supply voltages are 1.2 V for the core and 3.3 V for the inputs/outputs.

The OAs used are the model TLV2475N [17] by Texas Instruments. Their main characteristics are: CMOS Rail-To-Rail Input/Output, shutdown mode, input offset voltage: 2400μV (max), voltage amplification: 88 dB (min). From these parameters it can be deducted that, as commented in Section 3.5, the voltage which appears in the inverter terminal due to the finite gain is, at the most, 3.3 V/25119 = 0.13 mV, 20 times lower than offset voltage. The sensor array consists of eight rows and six columns. In addition, two rows and one column are used to measure the calibration resistors. Its values, along with the values of the capacitors of each of the columns of the array, are indicated in the following section for each of the experiments carried out.

## 5. Results and Discussion

The experiments carried out are detailed below:

### 5.1. Experiment 1

This experiment is carried out in order to analyse the performances of the Equation (6). The results are shown in Table 1.

**Table 1:** Accuracy data for Experiment 1.

Resistor (Ω)	$\bar{R}\;(\Omega )$	σ (Ω)	$\left|R-\bar{R}\right|(\Omega )$	$\left|R-\bar{R}\right|/R(\%)$	Max. Absolute Error (Ω)	Max. Relative Error (%)
556	571.25	0.26	15.25	2.743	16.24	2.92
5357.51	5157.26	1.41	200.25	3.738	207.20	3.87
10018.6	9196.84	2.48	821.76	8.202	830.23	8.29

Three resistors (560, 5357.51 and 10018.6 Ω) will be measured using 5350 Ω as nominal value for the calibration resistors. The other resistors of the array take the minimum value 560 Ω, this being the worst situation in terms of crosstalk in the array when evaluating the value of a resistor. The capacitors of each column of the array have a nominal value of 47 nF.

The results of $\bar{R}$ and σ have been obtained carrying out 500 measurements, whilst the errors of columns 4 and 5 of Table 1 show the worst case for the 500 measurements. The same procedure will be used for the other experiments of this section.

The maximum resistance *RH* permitted in accordance with the Equation (12) for an *RL* of 560 Ω is 870 Ω, a condition which is not met in any of the two *RL* resistors. Indeed it is verified that the results for the two last rows of the table show high absolute and relative errors. The same can be observed in the column which shows the systematic error (|R−R|). This happens even when using the *Zero* pin of the FPGA, as indicated in Section 2.

### 5.2. Experiment 2

In this case, the Equation (6) is once again used, but for a range of resistors (3.3 kΩ–10 kΩ), the minimum value for which is significantly higher than in the previous case. For the calibration resistors, 6.8 kΩ has been taken as the nominal value. The capacitors of each column of the array continue to be the same as in Experiment 1. In this case the value *RH* in accordance with Equation (12) is 5723.94 Ω, meaning we continue to have resistors which do not form part of the optimal measurement range.

Table 2 shows how both the systematic error and the maximum errors are reduced compared to Experiment 1. This confirms the discussion set out in Section 3.5 on the Equation (30), since in this Experiment the terms 2 and 4 of the right member of the equation are reduced proportionally more than term 1, having increased the minimum value of the array resistor.

**Table 2:** Accuracy data for Experiment 2.

Resistor (Ω)	$\bar{R}\;(\Omega )$	σ (Ω)	$\left|R-\bar{R}\right|(\Omega )$	$\left|R-\bar{R}\right|/R(\%)$	Max. Absolute Error (Ω)	Max. Relative Error (%)
3295.6	3294.39	2.02	1.21	0.037	17.33	0.53
3486.3	3485.68	2.00	0.62	0.018	17.81	0.51
3882.6	3882.18	1.63	0.42	0.011	17.28	0.45
4628.4	4628.11	2.43	0.29	0.006	23.27	0.50
5072.8	5073.11	2.99	0.31	0.006	25.26	0.50
5621.1	5621.82	3.68	0.72	0.013	29.76	0.53
6166.1	6166.54	4.40	0.44	0.007	31.63	0.51
6789.1	6789.51	3.84	0.41	0.006	31.08	0.46
7151.2	7152.23	4.97	1.03	0.014	41.49	0.58
7463.7	7465.13	4.87	1.43	0.019	41.99	0.56
8169.2	8171.69	5.22	2.49	0.030	44.53	0.55
9054.6	9057.01	6.62	2.41	0.027	51.64	0.57
9974.7	9975.08	8.45	0.38	0.004	58.48	0.59

Moreover, since the *RL* resistor is much greater than in Experiment 1, the *Zero* pin of the FPGA achieves a smaller value in the OA inverter input. In consequence, these two experiments show that the Equation (6) only applies in resistive sensors where *RL* > 3 kΩ.

### 5.3. Experiment 3

As observed in Experiment 1, the results are not as desired for the resistor ranges where *RL* takes lower values. For this reason, in this experiment the circuit of Figure 7 is implemented, allowing an increase in the range of resistors by modifying the row addressing. In this case there are two approximation methods which can be used: the Equation (22) which allows an increase in the range of resistors permitted, and Equation (44) which, in addition to increasing the range, eliminates the influence of the offset voltage on the EBT estimation. The range of resistors used in this case goes from 560 Ω to 3.3 kΩ. A value of 750 Ω has been chosen for the calibration resistors of row *c1* and the calibration column. 990 Ω has been taken as nominal value for the calibration resistors of row *c2*. The capacitors of each column of the array have a value of 330 nF.

As can be seen in Table 3, the errors, both systematic and relative, using the Equation (22) are much lower than those obtained in experiment 1. Moreover, the maximum resistor of the range used exceeds the maximum value permitted, *RH* = 2163 Ω, obtained from the Equation (16). Table 3 shows how, in greater *RH* values, these systematic errors are much higher than the others, although they are lower than those obtained for the same resistors of experiment 1.

**Table 3:** Accuracy data for *R_ij_* estimation by using Equation (22) R∈[556 Ω,3159 Ω].

Resistor (Ω)	$\bar{R}\;(\Omega )$	σ (Ω)	$\left|R-\bar{R}\right|(\Omega )$	$\left|R-\bar{R}\right|/R(\%)$	Max. Absolute Error (Ω)	Max. Relative Error (%)
556	555.08	0.38	0.92	0.165	2.52	0.45
678.5	677.81	0.52	0.69	0.102	2.67	0.39
747.2	745.85	0.60	1.35	0.181	3.81	0.51
864.2	863.61	0.84	0.59	0.068	3.91	0.45
1097	1096.46	1.01	0.52	0.047	4.33	0.39
1692.1	1692.95	1.90	0.85	0.050	9.90	0.58
2198	2194.15	3.26	3.85	0.175	18.95	0.86
2615.8	2611.20	4.38	4.40	0.168	28.10	1.07
3158.9	3151.54	5.96	7.36	0.233	25.16	0.80

Table 4 uses the same experimental data as those used in Table 3 but evaluated in accordance with the Equation (44). It can be seen that the systematic error is lower in this case, having considered the effects of offset. However it is observed that the maximum errors are reduced less than the systematic error. This is due to the fact that the Equation (44) uses six independent time measurements to carry out the estimation, whilst Equation (22), only needs four.

**Table 4:** Accuracy data for *R_ij_* estimation by using Equation (44) R∈[556 Ω,3159 Ω].

Resistor (Ω)	$\bar{R}\;(\Omega )$	σ (Ω)	$\left|R-\bar{R}\right|(\Omega )$	$\left|R-\bar{R}\right|/R(\%)$	Max. Absolute Error (Ω)	Max. Relative Error (%)
556	556.52	0.30	0.52	0.094	1.69	0.30
678.5	679.14	0.45	0.64	0.094	3.04	0.45
747.2	748.02	0.51	0.82	0.110	3.80	0.51
864.2	865.10	0.74	0.90	0.104	3.97	0.46
1097	1097.97	0.85	0.97	0.088	4.54	0.41
1692.1	1692.33	1.74	0.23	0.014	9.44	0.56
2198	2199.05	3.05	1.05	0.048	15.93	0.72
2615.8	2615.98	3.95	0.18	0.007	18.41	0.70
3158.9	3159.50	5.46	0.60	0.019	24.45	0.77

In this case, no increase is observed in the systematic errors for resistors above value *RH*.

### 5.4. Experiment 4

This experiment also uses the circuit of Figure 7, and the Equations (22) and (44) to calculate the value of *R_ij_*. The only change compared to experiment 3 is the range of resistors to study, 3.3–10 kΩ. A value of 10 kΩ has been chosen for the calibration resistors of row *c1* and the calibration column. 6.8 kΩ has been taken as nominal value for the calibration resistors of row *c2*. The capacitors of each column of the array have a value of 47 nF.

For this range the maximum resistor again exceeds the maximum value permitted, *RH* = 7560 Ω, obtained from the Equation (16). However, on this occasion, using Equation (22), as indicated in Table 5, does not show any important variations in the systematic and maximum errors for the resistors which exceed *RH*, as would be expected when increasing *RL* and for use of the *Zero* pin.

Again, Table 6 uses the same experimental data as those used in Table 5 but evaluated in accordance with Equation (44). The significant reduction can again be seen in the systematic and maximum errors compared to those obtained by Equation (22).

**Table 5:** Accuracy data for *R_ij_* estimation by using Equation (22) R∈[3296 Ω,9975 Ω].

Resistor (Ω)	$\bar{R}\;(\Omega )$	σ (Ω)	$\left|R-\bar{R}\right|(\Omega )$	$\left|R-\bar{R}\right|/R(\%)$	Max. Absolute Error (Ω)	Max. Relative Error (%)
3295.6	3278.86	1.89	16.74	0.508	25.88	0.79
3486.3	3468.56	1.97	17.74	0.509	31.41	0.90
3882.6	3863.51	2.07	19.09	0.492	30.52	0.79
4628.4	4605.27	2.59	23.13	0.500	45.03	0.97
5072.8	5046.77	3.47	26.03	0.513	44.81	0.88
5621.1	5592.11	3.44	28.99	0.516	49.21	0.88
6166.1	6132.44	3.92	33.66	0.546	53.75	0.87
6789.1	6751.06	4.34	38.04	0.560	66.99	0.99
7151.2	7114.69	4.71	36.51	0.511	70.85	0.99
7463.7	7427.34	6.22	36.36	0.487	89.98	1.21
8169.2	8125.95	7.29	43.25	0.529	86.61	1.06
9054.6	9010.28	8.88	44.32	0.489	98.50	1.09
9974.7	9924.17	9.80	50.53	0.507	110.99	1.11

**Table 6:** Accuracy data for *R_ij_* estimation by using Equation (44) R∈[3296 Ω,9975 Ω].

Resistor (Ω)	$\bar{R}\;(\Omega )$	σ (Ω)	$\left|R-\bar{R}\right|(\Omega )$	$\left|R-\bar{R}\right|/R(\%)$	Max. Absolute Error (Ω)	Max. Relative Error (%)
3295.6	3294.86	1.87	0.74	0.022	10.42	0.32
3486.3	3486.06	1.94	0.24	0.007	11.09	0.32
3882.6	3882.42	2.13	0.18	0.005	11.31	0.29
4628.4	4627.84	2.74	0.56	0.012	18.87	0.41
5072.8	5071.18	3.12	1.62	0.032	20.74	0.41
5621.1	5619.48	3.73	1.62	0.029	22.20	0.39
6166.1	6163.76	4.06	2.34	0.038	33.82	0.55
6789.1	6783.91	4.46	5.19	0.076	37.00	0.54
7151.2	7148.79	5.70	2.41	0.034	41.22	0.58
7463.7	7460.75	5.35	2.95	0.040	49.56	0.66
8169.2	8165.83	7.02	3.37	0.041	48.51	0.59
9054.6	9049.69	7.93	4.91	0.054	62.20	0.69
9974.7	9967.16	8.92	7.54	0.076	65.68	0.66

**Figure 8:**
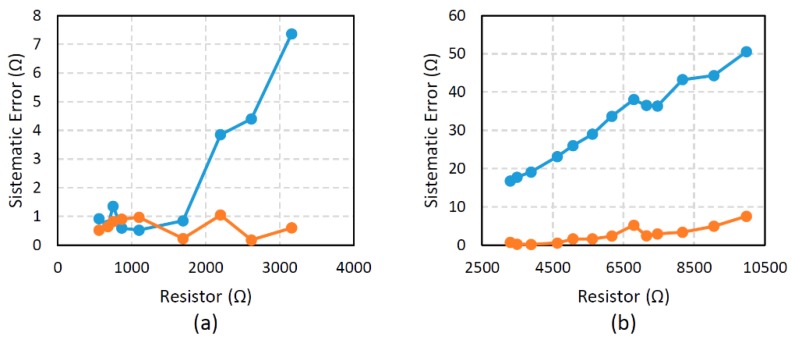
Systematic error in the two ranges of resistors used in Experiments 3 and 4. The results obtained by Equation (22) are shown in blue, and the results obtained by Equation (44) in red; (**a**) results from Experiment 3; (**b**) results from Experiment 4.

Figure 8 compares the results for Experiments 3 and 4. The errors are similar for the low resistor range for both methods, whilst values are estimated below *RH*. However, in the range of high resistors, the estimation based on Equation (44) provides better results in any case.

## 6. Conclusions/Outlook

This paper studies the use of a distribution of *M* rows and *N* columns for arrays of resistive sensors of different ranges and for many applications, for instance chemical, biological, robotics, *etc.* This distribution allows access, with a certain degree of parallelism (*M* simultaneous readings) to the information provided by the sensors. It also allows the use of simple conditioning circuits for analogue-digital conversion. The circuit, which enables a simple connection between the information of the resistive sensor and an FPGA as the converter element, comprises a series of *M* OAs with capacitive feedback. However, it presents certain limitations due to its inherent nature which reduce its functions. The causes of these limitations include: the appearance of crosstalk, the reduced range of resistors to measure (a function of the minimum resistor of the array), variability and difficulty in measuring parameters and important elements of the circuit (FPGA buffer resistors, the threshold voltages of these buffers, capacitors used in OA feedback) and, finally, the offset voltage and bias currents of the OAs. Different modiffications of the circuit and procedures have been proposed to mitigate each of these limitations. In order to check the effectiveness of the different proposed reading methods, a series of experiments have been carried out for a piezoresistive tactile sensor. Our final proposal achieves a maximum relative systematic error of 0.11% and a maximum relative error of 0.77% for an array with values in the range (556 Ω to 3159 Ω) and a maximum relative systematic error of 0.08% and a maximum relative error of 0.69% for an array with values in the range (3296 Ω to 9975 Ω). Future work will need to evaluate the influence of the finite gain of the OA on the performance of the circuit. It has been shown that in the design proposed in this document, this is much lower than the influence due to offset voltage. However, this may not be the case in other implementations.

## References

[1] Wu J., Wang L., Li J., Yu Z. (2011). A small size device using temperature sensor array. Chin. J. Sens. Actuators.

[2] Yang Y.-J., Cheng M.-Y., Shih S.-C., Huang X.-H., Tsao C.-M., Chang F.-Y., Fan K.-C. (2010). A 32 × 32 temperature and tactile sensing array using PI-copper films. Int. J. Adv. Manuf. Technol..

[3] Wang J., Chan S., Carlson R.R., Luo Y., Ge G., Ries R.S., Heath J.R., Tseng H.-R. (2004). Electrochemically fabricated polyaniline nanoframework electrode junctions that function as resistive sensors. Nano Lett..

[4] Depari A., Falasconi M., Flammini A., Marioli D., Rosa S., Sberveglieri G., Taroni A. (2007). A new low-cost electronic system to manage resistive sensors for gas detection. IEEE Sens. J..

[5] Shimojo M., Namiki A., Ishikawa M., Makino R., Mabuchi K. (2004). A tactile sensor sheet using pressure conductive rubber with electrical-wires stitched method. IEEE Sens. J..

[6] Castellanos-Ramos J., Navas-González R., Macicior H., Sikora T., Ochoteco E., Vidal-Verdú F. (2010). Tactile sensors based on conductive polymers. Microsyst. Technol..

[7] Kane B.J., Cutkosky M.R., Kovacs G.T.A. (2000). A traction stress sensor array for use in high-resolution robotic tactile imaging. J. Microelectromechan. Syst..

[8] Dahiya R.S., Metta G., Valle M., Adami A., Lorenzelli L. (2009). Piezoelectric oxide semiconductor field effect transistor touch sensing devices. Appl. Phys. Lett..

[9] Göger D., Worn H. A highly versatile and robust tactile sensing system. Proceedings of the 2007 IEEE Sensors.

[10] Wu J., Wang L., Li J. (2015). Design and Crosstalk Error Analysis of the Circuit for the 2-D Networked Resistive Sensor Array. IEEE Sens. J..

[11] Vidal-Verdú F., Barquero M.J., Castellanos-Ramos J., Navas-González R., Sánchez J.A., Serón J., García-Cerezo A. (2011). A Large Area Tactile Sensor Patch Based on Commercial Force Sensors. Sensors.

[12] Maldonado-Lopez R., Vidal-Verdu F., Linan G., Rodriguez-Vazquez A. (2009). Integrated Circuitry to Detect Slippage Inspired by Human Skin and Artificial Retinas. IEEE Trans. Circuits Syst. Regul. Pap..

[13] Vidal-Verdú F., Oballe-Peinado Ó., Sánchez-Durán J.A., Castellanos-Ramos J., Navas-González R. (2011). Three Realizations and Comparison of Hardware for Piezoresistive Tactile. Sensors.

[14] D’Alessio T. (1999). Measurement errors in the scanning of piezoresistive sensors arrays. Sens. Actuators Phys..

[15] Reverter F., Jordana J., Gasulla M., Pallàs-Areny R. (2005). Accuracy and resolution of direct resistive sensor-to-microcontroller interfaces. Sens. Actuators Phys..

[16] Xilinx, Inc. Spartan-3AN FPGA Family Data Sheet. http://www.xilinx.com/support/documentation/data_sheets/ds557.pdf.

[17] Texas Instruments, Inc. 600 uA/Ch 2.8 MHz Rail-to-Rail I/O High-Drive Op Amps with Shutdown. http://www.ti.com/lit/ds/symlink/tlv2475.pdf.

